# Association Between Area-Level Socioeconomic Deprivation and Diabetes Care Quality in US Primary Care Practices

**DOI:** 10.1001/jamanetworkopen.2021.38438

**Published:** 2021-12-29

**Authors:** Shaheen Shiraz Kurani, Michelle A. Lampman, Shealeigh A. Funni, Rachel E. Giblon, Jonathan W. Inselman, Nilay D. Shah, Summer Allen, David Rushlow, Rozalina G. McCoy

**Affiliations:** 1 Division of Health Care Delivery Research, Mayo Clinic, Rochester, Minnesota; 2 Mayo Clinic Robert D. and Patricia E. Kern Center for the Science of Health Care Delivery, Mayo Clinic, Rochester, Minnesota; 3 Department of Family Medicine, Mayo Clinic, Rochester, Minnesota; 4 Division of Community Internal Medicine, Department of Medicine, Mayo Clinic, Rochester, Minnesota

## Abstract

**Importance:**

Diabetes management operates under a complex interrelationship between behavioral, social, and economic factors that affect a patient's ability to self-manage and access care.

**Objective:**

To examine the association between 2 complementary area-based metrics, area deprivation index (ADI) score and rurality, and optimal diabetes care.

**Design, Setting, and Participants:**

This cross-sectional study analyzed the electronic health records of patients who were receiving care at any of the 75 Mayo Clinic or Mayo Clinic Health System primary care practices in Minnesota, Iowa, and Wisconsin in 2019. Participants were adults with diabetes aged 18 to 75 years. All data were abstracted and analyzed between June 1 and November 30, 2020.

**Main Outcomes and Measures:**

The primary outcome was the attainment of all 5 components of the D5 metric of optimal diabetes care: glycemic control (hemoglobin A_1c_ <8.0%), blood pressure (BP) control (systolic BP <140 mm Hg and diastolic BP <90 mm Hg), lipid control (use of statin therapy according to recommended guidelines), aspirin use (for patients with ischemic vascular disease), and no tobacco use. The proportion of patients receiving optimal diabetes care was calculated as a function of block group–level ADI score (a composite measure of 17 US Census indicators) and zip code–level rurality (calculated using Rural-Urban Commuting Area codes). Odds of achieving the D5 metric and its components were assessed using logistic regression that was adjusted for demographic characteristics, coronary artery disease history, and primary care team specialty.

**Results:**

Among the 31 934 patients included in the study (mean [SD] age, 59 [11.7] years; 17 645 men [55.3%]), 13 138 (41.1%) achieved the D5 metric of optimal diabetes care. Overall, 4090 patients (12.8%) resided in the least deprived quintile (quintile 1) of block groups and 1614 (5.1%) lived in the most deprived quintile (quintile 5), while 9193 patients (28.8%) lived in rural areas and 2299 (7.2%) in highly rural areas. The odds of meeting the D5 metric were lower for individuals residing in quintile 5 vs quintile 1 block groups (odds ratio [OR], 0.72; 95% CI, 0.67-0.78). Patients residing in rural (OR, 0.84; 95% CI, 0.73-0.97) and highly rural (OR, 0.81; 95% CI, 0.72-0.91) zip codes were also less likely to attain the D5 metric compared with those in urban areas.

**Conclusions and Relevance:**

This cross-sectional study found that patients living in more deprived and rural areas were significantly less likely to attain high-quality diabetes care compared with those living in less deprived and urban areas. The results call for geographically targeted population health management efforts by health systems, public health agencies, and payers.

## Key Points

**Question:**

What is the association between area deprivation index, rurality, and diabetes care quality?

**Findings:**

In this cross-sectional study of 31 934 adult patients with diabetes receiving care at primary care practices across 3 US states, census block group–level area deprivation index score and zip code–level rurality were significantly associated with diabetes care quality.

**Meaning:**

Findings from this study showed that adult patients with diabetes who lived in more deprived and rural areas were significantly less likely to attain high-quality diabetes care compared with those in less deprived and urban areas, calling for geographically targeted efforts to improve care quality and health outcomes for disadvantaged populations.

## Teaser

This cross-sectional study examines the association of socioeconomic, geographic, and behavioral factors with the quality of diabetes management for adults.

## Introduction

In the US, 34.1 million people, or 13% of the population, are living with diabetes.^1^ Diabetes and its complications are associated with poor health,^2^ shortened life expectancy,^3,4^ and impaired quality of life.^5,6^ In 2017 alone, $237 billion was spent in the US on direct medical costs related to this disease.^2^ The burden of diabetes and its complications is disproportionately larger for racial and ethnic minority groups, low-income individuals, and rural residents.^7,8,9^ Factors in high rates of diabetes complications in these underserved populations include suboptimal control of hyperglycemia and other cardiovascular disease risk factors,^10^ including hypertension, dyslipidemia, and tobacco smoking.^8,11^ To improve the quality of diabetes care, health systems and payers rely on publicly reported metrics to evaluate care delivery, identify opportunities for improvement, and support pay-for-performance reimbursement. However, achieving equity in diabetes care also requires these metrics to be leveraged to identify populations and regions with lagging health outcomes. When such data are available in real time, quality metrics can generate actionable information for clinicians, health systems, public health agencies, and payers on subgroups of patients in need of additional support and targeted interventions.

Diabetes management is challenging because of the complex interactions among multiple behavioral, social, and economic factors in a patient's ability to self-manage and access necessary care. Epidemiologic studies on the prevalence of diabetes have found substantial disparities across each of the 5 constructs that are most commonly represented in the social determinants of health framework^12^: economic stability,^13,14^ educational level,^15^ neighborhood and built environment,^16^ health and health care,^17,18^ and social and community context.^8,19^ However, most studies examining disparities in diabetes management and outcomes have focused on select socioeconomic conditions without capturing the full complexity of a patient’s situation, such as poverty and social or environmental context.^8^

One measure that captures area-level social determinants of health is the area deprivation index (ADI). The ADI score is a composite indicator of area-based socioeconomic disadvantages in the 4 domains outside of the strictly defined health care setting: income, housing, employment, and education. Previous studies have revealed disparities in cancer screening,^20^ opioid use,^21^ and drug-related mortality^21^ as a function of the ADI score. However, evidence is scarce on the association between area-level deprivation (as captured by a multifaceted indicator of social determinants of health, such as the ADI) and diabetes care quality. Even less contemporary evidence is available about the potential differences in diabetes care quality between rural and urban areas as well as about the potential intersection between rurality and deprivation.

To address these critical knowledge gaps and demonstrate how electronic health record (EHR) data can be the basis of real-time evaluations of care quality and equity in routine clinical practice, we examined the associations between 2 complementary area-based metrics, ADI score and rurality, and optimal diabetes care as defined by a quality measure that is frequently used in population health management. Specifically, in this study, we focused on the D5 composite quality metric of optimal diabetes care, a measure that was developed and is tracked by Minnesota Community Measures and is used by health care organizations and health care practices in Minnesota. Minnesota Community Measures defined optimal diabetes care as having the following outcome: a hemoglobin A_1c_ (HbA_1c_) level that is less than 8.0%; a blood pressure (BP) reading that is less than 140/90 mm Hg; statin use that is appropriate for the patient’s age, low-density lipoprotein cholesterol (LDL-C) level, and history of cardiovascular disease; aspirin use that is appropriate in the setting of ischemic vascular disease; and abstinence from tobacco use.^22^ Public reporting of the D5 metric performance is mandatory for all health care practices in Minnesota and is used to guide performance-based reimbursement. Understanding the association between these components of optimal diabetes care and the area-based metrics of ADI score and rurality can help inform the multidisciplinary and multifaceted efforts to improve both the quality and equity of diabetes care.

## Methods

This cross-sectional analysis of EHR data included adults aged 18 years or older who received primary care at any of the 75 Mayo Clinic and Mayo Clinic Health System primary care practices across 54 communities (towns or cities) in Minnesota, Iowa, and Wisconsin. All data were abstracted and analyzed between June 1 and November 30, 2020. The study was approved by the Mayo Clinic Institutional Review Board, which waived the requirement for informed consent because the study was deemed to pose minimal risk. We followed the Strengthening the Reporting of Observational Studies in Epidemiology (STROBE) reporting guideline.^23^

### Study Population

The primary analysis was conducted among patients aged 18 to 75 years who had an established diagnosis of diabetes as of December 31, 2019, and who were empaneled to a Mayo Clinic or Mayo Clinic Health System primary care practice in Minnesota, Iowa, and Wisconsin. Patients with a diabetes diagnosis during a clinical encounter were identified using *International Statistical Classification of Diseases, Tenth Revision, Clinical Modification* codes. The age subgroup was selected according to the eligibility criteria for the quality measure reported to Minnesota Community Measures.^22^

Each patient’s primary address was linked to a block group using the Census Geocoder, a program for obtaining latitude and longitude points for each address.^24^ The coordinates were spatially joined to a TIGER/Line block group Shapefile in ArcMap 10.7 (Esri). We excluded patients without a valid zip code and patients whose address could not be geocoded with a match score greater than 60, a measure that represents how reliably the patient address matched a candidate in the reference data. Patients were assigned a Rural-Urban Commuting Area (RUCA) code based on the zip code of their residence.^25,26^

### Outcomes

The primary outcome was the attainment of all 5 components of the D5 metric of optimal diabetes care,^22^ as recorded in the EHR in 2019. The EHR was used to ascertain the performance on the D5 metric not only in this research but also in clinical practice for reporting to Minnesota Community Measures. Achievement of the D5 metric components means showing glycemic control (HbA_1c_ <8.0%), BP control (systolic BP <140 mm Hg and diastolic BP <90 mm Hg), lipid control (use of statin therapy according to recommended guidelines), aspirin use (for patients with ischemic vascular disease), and confirmed (self-reported) no tobacco use.^27^

Guideline-recommended statin use is dependent on age, LDL-C level within the past 5 years, and history of cardiovascular disease. Specifically, the following criteria need to be present for the statin indicator to be successfully met: 18 to 20 years of age regardless of LDL-C level (ie, they are not required to be treated with a statin but meet this metric regardless); 21 to 39 years of age with either an LDL-C level that is less than 190 mg/dL or treatment with a statin; 40 to 75 years of age with either an LDL-C level that is less than 70 mg/dL or treatment with a statin; and any age with a history of vascular disease and either an LDL-C level that is less than 40 mg/dL or treatment with a statin (to convert LDL-C level to millimoles per liter, multiply by 0.0259). Patients with a documented contraindication (eg, pregnancy), intolerance, or allergy to statin therapy automatically meet the statin measure without the requirement for a documented statin prescription or particular LDL-C level. An active prescription for daily aspirin use is required only for individuals with established ischemic vascular disease and no documented allergy, intolerance, or contraindication; all patients without ischemic vascular disease or patients with allergy, intolerance, or contraindication automatically meet this measure even without an aspirin prescription.

The secondary outcomes were meeting each subcriterion of the D5 metric. The exception was aspirin use because nearly all (99.3%) patients in the study population met this metric.

At Mayo Clinic Health System, performance on the D5 metric is calculated and internally shared at the regional, clinic, care team, and individual clinician levels. Regions and clinics function semiautonomously with regional approaches to care quality, including for diabetes. Each practice within the Mayo Clinic Health System has autonomy to implement local quality improvement initiatives, with no standard approach across the health system.

### Area Deprivation Index

We examined achieving the D5 metric (primary outcome) and individually meeting the 4 nonaspirin subcriteria of the D5 metric (secondary outcomes) as a function of the block group ADI score. Block group–level information that was necessary for ADI score derivation was obtained from the 5-year American Community Survey, an annual survey conducted by the US Census Bureau that provides population-level estimates that are representative of the noninstitutionalized US population.^28^ In-depth survey methods are found on the US Census Bureau website.^28,29^

We used 17 block-group indicators, representing income, employment, housing, and education, to compute ADI scores for all US Census block groups.^30,31,32,33^ All block groups were ranked by ADI scores. Each block group was assigned to a quintile of ADI scores from the least deprived 20% of block groups (quintile 1) to the most deprived 20% of block groups (quintile 5). Weights that were assigned to each variable in the ADI are presented in the eTable in the Supplement. A geographic hot spot map of block group ADI scores in Minnesota, Iowa, and Wisconsin (n = 11 230) was created and has been previously described.^20^

### Independent Variables

Rurality was ascertained from patient zip codes to identify corresponding RUCA codes. Based on published definitions, the RUCA codes classified areas as urban (1.0, 1.1, 2.0, 2.1, 3.0, 4.1, 5.1, 7.1, 8.1, 10.1), rural (4.0, 4.2, 5.0, 5.2, 6.0, 6.1, 7.0, 7.2, 7.3, 7.4, 8.0, 8.2, 8.3, 8.4, 9.0, 9.1, 9.2), or highly rural (10.0, 10.2, 10.3, 10.4, 10.5, 10.6).^34^ Zip code–level RUCA codes were used as individual risk factors to make patient-level inferences, given that block group–level RUCA codes were not available through the US Department of Agriculture.^25,26^ Detailed information regarding RUCA codes can be found on the US Department of Agriculture website.^26,34^

Patient demographic characteristics (sex, race and ethnicity, and age), history of coronary artery disease, and primary care team specialty (internal medicine, family medicine, or other) were ascertained from the EHR. Race and ethnicity were classified as White or racial and ethnic minority group. This group comprised African, African American, American Indian/Alaskan Native, Asian (including subcategories that were based on country of origin such as Cambodian, Chinese, Filipino, Indian, Japanese, Korean, Laotian, Pakistani, Taiwanese, Thai, and Vietnamese), Black, Caribbean Black, Native Hawaii/Pacific Islander, and Samoan; those who did not provide race and ethnicity, responded with “other,” or identified 2 or more affiliations were also included. These categories were combined because of the small number of patients representing each category, which would preclude analyses. Race and ethnicity were self-reported by the patient during registration and documented in the EHR.

### Statistical Analysis

We calculated overall frequencies (percentages) and means (SDs) for baseline patient characteristics. We used multivariable logistic regression to examine the associations between ADI score, rurality, and the outcomes (ie, primary outcome of attaining the D5 metric and secondary outcomes of meeting the nonaspirin subcriteria of the D5 metric). Independent variables in the models included ADI scores by quintile, rural status, age, race and ethnicity, sex, history of coronary artery disease, and care team specialty. We used Huber-White robust SEs clustered at the practice level to adjust SEs for variation in care delivery, resources, and recommendations across the primary care clinics. Analyses were conducted to test for an interaction between ADI score and rurality, and no interaction was found.

A 2-sided *P* = .05 was used as the threshold of statistical significance. Analyses were conducted using SAS, version 9.4 (SAS Institute Inc), and Stata, version 15.1 (StataCorp LLC).

## Results

The study cohort comprised 31 934 patients (17 645 men [55.3%] and 14 289 women [44.8%]) who were eligible for the D5 metric assessment. These patients had a mean (SD) age of 59 (11.7) years and were predominantly White individuals (n = 29 180 [91.4%]). Overall, 1614 patients (5.1%) lived in the most deprived quintile (quintile 5) and 4090 (12.8%) lived in the least deprived quintile (quintile 1); 9193 patients (28.8%) lived in rural areas and 2299 (7.2%) lived in highly rural areas (Table 1).

**Table 1.** Patient Characteristics at the Start of the Measurement Year

Characteristic	No. (%)		
	Patients who attained the D5 metric (n = 13 138)	Patients who did not attain the D5 metric (n = 18 796)	All patients (n = 31 934)
Age, y			
Mean (SD)	57.8 (12.0)	61.8 (10.9)	59 (11.7)
18-44	1070 (8.1)	2671 (14.2)	3741 (11.7)
45-64	5505 (41.9)	9700 (51.6)	15 205 (47.6)
65-75	6536 (49.9)	6425 (34.2)	12 988 (40.7)
Sex			
Female	6119 (46.6)	8170 (43.5)	14 289 (44.8)
Male	7019 (53.4)	10 626 (56.5)	17 645 (55.3)
Race and ethnicity^a^			
Racial and ethnic minority group^b^	948 (7.2)	1806 (9.6)	2754 (8.6)
White	12 190 (92.8)	16 990 (90.4)	29 180 (91.4)
Coronary artery disease			
Present or previous diagnosis	2096 (16.0)	2544 (13.5)	4640 (14.5)
No history	11 042 (84.1)	16 252 (86.5)	27 294 (85.5)
ADI score quintile			
1 (least deprived)	1793 (13.6)	2297 (12.2)	4090 (12.8)
2	4669 (35.5)	6109 (32.5)	10 778 (33.6)
3	3971 (30.2)	5720 (30.4)	9691 (30.4)
4	2163 (16.5)	3598 (19.1)	5761 (18.0)
5	542 (4.1)	1072 (5.7)	1614 (5.1)
Rurality			
Urban	8771 (66.8)	11 671 (62.1)	20 442 (64.0)
Rural	3492 (26.5)	5701 (30.3)	9193 (28.8)
Highly rural	875 (6.7)	1424 (7.6)	2299 (7.2)
Practice specialty			
Internal medicine	3311 (25.2)	4521 (24.1)	7832 (24.5)
Family medicine	9413 (71.6)	13 779 (73.3)	23 192 (72.6)
Other^c^	414 (3.2)	496 (2.6)	910 (2.9)
D5 metric components			
Glycemic control	13 138 (100.0)	7833 (41.7)	20 971 (65.7)
Blood pressure control	13 138 (100.0)	11 188 (60.0)	24 326 (76.2)
Lipid control	13 138 (100.0)	14 581 (77.6)	27 719 (86.8)
No tobacco use	13 138 (100.0)	12 820 (68.2)	25 958 (81.3)
Aspirin use	13 138 (100.0)	18 566 (98.8)	31 704 (99.3)

Abbreviation: ADI, area deprivation index.

^a^ Race and ethnicity were self-reported by the patient and documented in the electronic health record.

^b^ This group comprised African, African American, American Indian/Alaskan Native, Asian (including subcategories that were based on country of origin such as Cambodian, Chinese, Filipino, Indian, Japanese, Korean, Laotian, Pakistani, Taiwanese, Thai, and Vietnamese), Black, Caribbean Black, Native Hawaii/Pacific Islander, and Samoan. Those who did not provide race and ethnicity, responded with “other,” or identified 2 or more affiliations were also included. These categories were combined because of the small number of patients representing each category, which would preclude analyses.

^c^ Other included mixed team, nursing home, pediatric resident, pediatrics, and women’s health.

Overall, 13 138 of 31 934 patients (41.1%) achieved the composite D5 metric of optimal diabetes care. Patients who attained the D5 metric compared with those who did not (n = 18 796) often were older (aged 65-75 years: 6536 [49.9%] vs 6425 [34.2%]), women (6119 [46.6%] vs 8170 [43.5%]), White individuals (12 190 [92.8%] vs 16 990 [90.4%]), and those who resided in less deprived and less rural areas. Patients who met the D5 metric had a slightly higher mean (SD) number of clinician visits for primary care, endocrinology, and diabetes education compared with those who did not meet the D5 metric (5.00 [5.68] vs 4.32 [6.00]).

### Association of ADI Score With the D5 Metric

Block group–level ADI score was associated with achieving the composite D5 metric and the individual glycemic control and no tobacco use components. As shown in the Figure, the adjusted probability of attaining the D5 metric decreased incrementally across ADI score quintiles (Table 2). The odds of meeting the D5 metric goals were approximately 28% lower for individuals who were living in quintile 5 vs those living in quintile 1 (odds ratio [OR], 0.72; 95% CI, 0.67-0.78).

**Figure.**
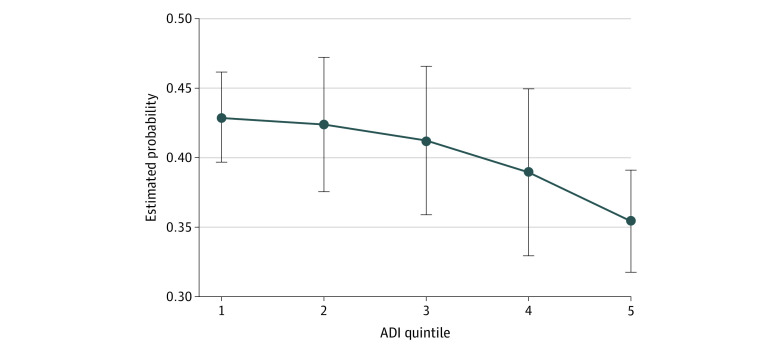
Estimated Probability of Attaining the D5 Metric by Area Deprivation Index (ADI) Score Quintile Error bars represent 95% CIs. Estimated probabilities were adjusted for the covariates shown in Table 2.

**Table 2.** Association Between Area Deprivation Index Score, Rurality, and Quality of Diabetes Care^a^

	OR (95% CI)				
	All D5 metric components	Glycemic control	Blood pressure control	Lipid control	No tobacco use
ADI score quintile					
1 (least deprived)	1 [Referent]	[Referent]	[Referent]	[Referent]	[Referent]
2	0.98 (0.91-1.05)	0.99 (0.86-1.13)	1.12 (1.02-1.22)^b^	0.94 (0.86-1.02)	0.70 (0.60-0.80)^b^
3	0.93 (0.84-1.04)	0.95 (0.83-1.09)	1.19 (1.03-1.38)^b^	0.99 (0.87-1.14)	0.54 (0.48-0.61)^b^
4	0.84 (0.74-0.97)^b^	0.85 (0.74-0.98)^b^	1.17 (0.99-1.39)	0.88 (0.78-1.00)^b^	0.46 (0.39-0.55)^b^
5	0.72 (0.67-0.78)^b^	0.78 (0.74-0.84)^b^	1.00 (0.82-1.21)	1.12 (1.03-1.22)^b^	0.38 (0.31-0.48)^b^
Rurality					
Urban	[Referent]	[Referent]	[Referent]	[Referent]	[Referent]
Rural	0.84 (0.73-0.97)^b^	0.87 (0.75-1.00)	0.82 (0.67-1.01)	0.88 (0.81-0.95)^b^	0.95 (0.88-1.03)
Highly rural	0.81 (0.72-0.91)^b^	0.90 (0.84-0.98)^b^	0.93 (0.75-1.16)	0.83 (0.77-0.89)^b^	0.92 (0.78-1.08)
Age, y					
18-44	[Referent]	[Referent]	[Referent]	[Referent]	[Referent]
45-64	1.41 (1.27-1.57)^b^	1.65 (1.48-1.84)^b^	0.95 (0.90-1.01)	0.94 (0.81-1.08)	1.49 (1.39-1.59)^b^
65-75	2.49 (2.25-2.76)^b^	3.00 (2.51-3.60)^b^	1.03 (0.93-1.15)	1.74 (1.51-2.00)^b^	3.33 (3.20-3.48)^b^
Sex					
Female	[Referent]	[Referent]	[Referent]	[Referent]	[Referent]
Male	0.87 (0.83-0.92)^b^	0.87 (0.84-0.90)^b^	0.84 (0.80-0.89)^b^	1.10 (0.98-1.24)	0.69 (0.63-0.75)^b^
Race and ethnicity^c^					
Racial and ethnic minority group^d^	0.85 (0.71-1.02)	0.72 (0.61-0.84)^b^	0.83 (0.72-0.95)^b^	0.76 (0.67-0.85)^b^	1.18 (1.02-1.36)^b^
White	[Referent]	[Referent]	[Referent]	[Referent]	[Referent]
Coronary artery disease					
No history	[Referent]	[Referent]	[Referent]	[Referent]	[Referent]
Present or previous diagnosis	1.04 (0.98-1.09)	1.01 (0.90-1.14)	1.23 (1.18-1.27)^b^	3.52 (3.02-4.10)^b^	0.76 (0.74-0.78)^b^
Practice specialty					
Internal medicine	[Referent]	[Referent]	[Referent]	[Referent]	[Referent]
Family medicine	1.04 (0.94-1.16)	1.08 (0.96-1.21)	1.17 (0.87-1.58)	0.89 (0.73-1.09)	0.79 (0.73-0.85)^b^
Other^e^	1.16 (0.83-1.62)	1.12 (0.93-1.34)	1.24 (0.97-1.59)	1.08 (0.66-1.76)	0.96 (0.74-1.26)

Abbreviations: ADI, area deprivation index; OR, odds ratio.

^a^ Multivariable logistic regression analysis examined the association between ADI score, rurality, and achieving the D5 metric components of optimal diabetes care (primary outcome) and meeting the subcriteria of the D5 metric (secondary outcomes) after adjusting for the patient-level demographic and clinical factors in this table.

^b^ *P* < .05.

^c^ Race and ethnicity were self-reported by the patient and documented in the electronic health record.

^d^ This group comprised African, African American, American Indian/Alaskan Native, Asian (including subcategories that were based on country of origin such as Cambodian, Chinese, Filipino, Indian, Japanese, Korean, Laotian, Pakistani, Taiwanese, Thai, and Vietnamese), Black, Caribbean Black, Native Hawaii/Pacific Islander, and Samoan. Those who did not provide race and ethnicity, responded with “other,” or identified 2 or more affiliations were also included. These categories were combined because of the small number of patients representing each category, which would preclude analyses.

^e^ Other included mixed team, nursing home, pediatric resident, pediatrics, and women’s health.

Within the D5 metric, the most variability was observed for the glycemic control and no tobacco use components. The odds of achieving an HbA_1c_ level that was less than 8.0% were 22% lower for individuals living in quintile 5 vs quintile 1 (OR, 0.78; 95% CI, 0.74-0.84). The odds of meeting the no tobacco use metric decreased progressively as deprivation increased, with patients residing in quintile 5 being 62% less likely to achieve no tobacco use compared with patients living in quintile 1 (OR, 0.38; 95% CI, 0.31-0.48). The odds of meeting the BP control component were highest in patients residing in quintile 2 (OR, 1.12; 95% CI, 1.02-1.22) and quintile 3 (OR, 1.19; 95% CI, 1.03-1.38) compared with those residing in quintile 1 block groups. For lipid control, patients in quintile 5 block groups were significantly more likely to meet the component compared with those in quintile 1 block groups (OR, 1.12; 95% CI, 1.03-1.22).

### Association of Rurality With the D5 Metric

Patients residing in rural zip codes were 16% less likely to attain the D5 metric compared with those living in urban zip codes (OR, 0.84; 95% CI, 0.73-0.97) (Table 2). Patients residing in highly rural zip codes were 19% less likely to achieve the composite D5 metric (OR, 0.81; 95% CI, 0.72-0.91) and were 10% less likely to achieve glycemic control (OR, 0.90; 95% CI, 0.84-0.98) than those living in urban areas. Patients from both rural (OR, 0.88; 95% CI, 0.81-0.95) and highly rural (OR, 0.83; 95% CI, 0.77-0.89) zip codes were less likely to achieve lipid control.

Blood pressure control and no tobacco use were not associated with rurality. We also tested for an interaction between ADI score and rurality, which was not present; hence, the interaction terms were not included in the final model.

### Patient-Level Factors Associated With Diabetes Care Quality

Patients from racial and ethnic minority groups with diabetes had lower odds of meeting the glycemic, lipid, and BP control components of the D5 metric compared with White patients, although no significant association was found between race and ethnicity and the D5 metric (Table 2). Older patients were more likely to attain the D5 metric (age 45-64 years: OR, 1.42 [95% CI, 1.27-1.57]; age 65-75 years: OR, 2.49 [95% CI, 2.25-2.76] vs age 18-44 years) as well as the glycemic control (age 45-64 years: OR, 1.65 [95% CI, 1.48-1.84]; age 65-75 years: OR, 3.00 [95% CI, 2.51-3.60] vs age 18-44 years), lipid control (age 65-75 years: OR, 1.74 [95% CI, 1.51-2.00] vs age 18-44 years), and no tobacco use (age 45-64 years: OR, 1.49 [95% CI, 1.39-1.59]; age 65-75 years: OR, 3.33 [95% CI, 3.20-3.48] vs age 18-44 years) components. Patients with coronary artery disease were more likely to achieve BP control (OR, 1.23; 95% CI, 1.18-1.27) and lipid control (OR, 3.52; 95% CI, 3.02-4.10) but less likely to achieve no tobacco use (OR, 0.76; 95% CI, 0.74-0.78). Men had lower odds of attaining the complete and individual D5 metric components, with the exception of lipid control, which showed no significant association with sex.

## Discussion

Population health strategies to improve the quality and equity of diabetes management and health outcomes require real-time tracking of performance and identification of populations in need of focused interventions. Geographic areas with gaps in care quality may benefit from targeted allocation of resources to address the factors associated with suboptimal care delivery and health outcomes. To identify areas of suboptimal diabetes care, we used clinical data from 75 primary care practices across 3 states that were linked to publicly available geographic information from the US Census Bureau. We found that adult patients with diabetes who lived in more deprived and in rural areas were significantly less likely to achieve high-quality diabetes care, as measured by the D5 metric, compared with patients who lived in less deprived and urban areas. These findings not only underscore the implications of area-level social determinants of health for diabetes care quality but also signal the need for geographically targeted population health management efforts by health systems, public health agencies, and payers.

People living in socioeconomically deprived areas face multiple obstacles to optimal diabetes care. Rates of type 2 diabetes are substantially higher in neighborhoods that are characterized by lower income, lower educational attainment, single-parent households, and crowded housing.^27,35^ Individuals living in areas of greater deprivation often have fewer financial resources, lower health literacy, greater comorbidity burden, and higher food insecurity.^8^ In addition, deprived neighborhoods are associated with an increased rate of obesity and decreased physical activity.^36^ These spatial social determinants of health are associated with the risk of developing diabetes, barriers to optimal self-management, and greater risk of diabetes-related complications. Clinics, health systems, payers, and public health agencies can, therefore, improve diabetes management and health outcomes in neighborhoods by addressing some of the structural factors that stymie optimal care.^8^ For example, health systems can increase the availability of social services in clinics; educate health care staff on screening for social determinants of health; bring medical education and outreach to trusted community centers; support food banks with nutritious food options^37^ and integrated diabetes self-management education or support; and partner with municipalities and public health agencies to build community centers,^38^ parks,^38^ and supportive housing.^39^ Payers can reduce patient cost-sharing responsibilities for diabetes-related care (eg, medications, durable medical equipment and supplies, and appointments),^40,41^ reimburse social services and support programs (such as community health worker and community paramedic programs),^42^ and lower insurance premiums for patients who adhere to treatment recommendations and improve their health.

Rural areas have a 17% higher prevalence of diabetes than urban areas.^43,44^ Yet few studies have examined the quality of diabetes care in rural communities, mostly because available data are scarce and sample sizes are small.^45^ The present study involved a large primary care population across 75 clinics in 3 Midwestern states, allowing the examination of both urban and rural settings. We found that patients in rural areas were significantly less likely to receive high-quality diabetes care, as measured by the D5 metric, reinforcing the gaps in access to care and care quality in rural communities.^46^ The role of rurality in diabetes care quality was independent of the ADI score, signifying the additional constraints on optimal diabetes care posed by rurality. Prior research has found that rural residents had higher rates of being uninsured or underinsured and had fewer resources, both medical and nonmedical, to optimally care for their diabetes. This lack of resources included fewer primary care and specialist clinicians as well as less access to diabetes education, exercise facilities, sidewalks for walking, and grocery stores with affordable produce.^8,43,46^ Patients who lived in rural areas were often unable to order glucose test strips^47,48^ and may forgo routine screening appointments.^49^ All these factors may contribute to the worse quality of diabetes care observed in the current study among patients living in rural and highly rural areas.

These geographic disparities that affect rural populations present major challenges for patients with diabetes and point to the need for geographically tailored interventions that take into consideration the specific resources available in rural sites. Implementing telemedicine capabilities or mobile telehealth units for diabetes care in difficult-to-reach and underserved areas may address access-related issues. However, telemedicine would not be a viable option for patients without broadband internet, which is frequently a barrier in highly rural areas. Community health workers,^8^ community paramedics,^50,51^ and endocrinology experts in rural primary care practices (eg, Endo ECHO model)^52,53^ can help address some of the gaps in rural and remote areas.

Components of the D5 metric that were associated with the ADI score were glycemic control and no tobacco use, with residents of the most deprived areas being 22% less likely to achieve an HbA_1c_ level that was less than 8.0% and 62% less likely to not use tobacco compared with residents of the least deprived areas. Both of these metrics were outcome indicators that relied heavily on individual behavior change, medication adherence (for glycemic control), and self-efficacy. This finding contrasts with the lipid control and aspirin use metrics, which were process indicators that were achieved simply by clinicians prescribing the appropriate pharmacotherapy. Although an outcome indicator of lipid control would face the same challenges as the glycemic control, BP control, and no tobacco use components, the process indicator of having an active statin prescription on file (regardless of whether that statin prescription was filled or what LDL-C level was achieved) was easier to meet. Lifestyle therapy (ie, medical nutrition therapy) is a core component of successful glycemic control, and patients in socioeconomically deprived areas are more likely to experience food insecurity, lack of nutritious food choices, and inadequate safe spaces for physical activity. In addition, many glucose-lowering medications are expensive, making it challenging for low-income patients to access and fully adhere to clinically preferred treatment regimens. Previous studies found that access to diabetes self-management education in low-income and rural areas was inadequate,^54,55^ which was associated with greater probability of forgoing medical care.^56,57^ All of these structural factors need to be addressed to improve diabetes care quality in socioeconomically deprived areas.

Similarly, although the prevalence of smoking has declined in the US over the past 60 years, tobacco use remains concentrated among low-income and other socioeconomically disadvantaged populations.^58^ Therefore, focused smoking and other tobacco use–cessation interventions are needed that are tailored to residents of socioeconomically deprived areas. Such efforts include referral pathways for free smoking-cessation programs^59^; expanded access to smoking-cessation counseling and medication benefits; antismoking media, social media, and community campaigns^60,61,62,63^; and smoking bans in public housing.^64^

Blood pressure control is another outcome measure but is generally more amenable to pharmacologic intervention than glycemic control. Still, it is unclear why patients in both the least and the most deprived ADI score quintiles were less likely to achieve the BP control metric than patients in the middle quintiles (which is a finding that is consistent with results of previous work).^65^ A recent population-based study of patients with diabetes across the US found that patients with both high and low incomes, according to the federal poverty level, were less likely to be treated with antihypertensive medications than patients with an intermediate income level,^66^ suggesting that those in both extremes of income may be more likely to be undertreated for their hypertension. However, in other studies, no significant association was found between BP control and either income or rurality.^67^ Thus, the reasons behind better BP control among patients residing in lower-income areas will need to be explored in future research.

Independent of area-level factors (ie, ADI score and rurality), patients from racial and ethnic minority groups were significantly less likely to attain high-quality diabetes care. Our findings build on robust literature that confirmed racial disparities in diabetes-related health outcomes, including higher rates of both acute (ie, severe hypoglycemia^68,69,70,71^ and hyperglycemia or ketoacidosis^68,69^) and chronic (ie, kidney failure,^72^ amputation,^72^ and cardiovascular disease^72^) complications among Black patients with diabetes compared with White patients. As a result of historical and contemporary residential segregation, predominantly Black neighborhoods are more likely to be characterized by food deserts, fewer recreational facilities, environmental chemicals and toxins, and lower-quality housing vs predominantly White neighborhoods.^8,73^ These structural barriers have all been found to be associated with diabetes prevalence and health outcomes. This finding signals a need for policy makers to tighten environmental safety regulations in areas of concentrated poverty, which are most often disadvantaged and multiracial and multiethnic neighborhoods, as well as to improve insurance access and coverage for those who are unable to afford it.

Improving health equity and eliminating health disparities are urgent national priorities.^74^ We believe this study provides a framework for evaluating diabetes care quality and equity through the lens of geographic disparities, yielding rapidly actionable information for health systems, policy makers, and payers to drive innovation and improvement in underserved areas.

### Strengths and Limitations

This study has some strengths. To our knowledge, this study was the first multisite investigation into area-level variation in diabetes care quality that focused on socioeconomic deprivation and rurality across a diverse geographic area in 3 states. The study is strengthened by granular patient-level data that allowed us to contextualize geospatial disparities at the census block-group level with the patient’s clinical context and care.

This study also has some limitations. Because we used survey data when calculating ADI scores, the calculations are susceptible to nonresponse bias. In addition, the findings may not be generalizable to other settings because of the lower representation of racial and ethnic minority groups in the included clinical sites than in the general US population. Nevertheless, the study population was representative of the upper Midwest and rural communities across the country.^75^ Although we cannot draw causal inferences from this observational study, the findings show how readily available EHR data and tools can be used to track not only the quality but also the equity of chronic disease care. Such data, when available in real time, can inform and support interventions to improve the health of all people with diabetes.

## Conclusions

This cross-sectional study found that adult patients with diabetes in areas that were more socioeconomically deprived and rural were significantly less likely to attain the D5 metric of optimal diabetes care compared with patients who lived in less deprived and urban areas. Geographically targeted population health management efforts by health systems, public health agencies, and payers are needed to improve the care quality and health outcomes for disadvantaged populations.

## Notes

Supplement. **eTable.** American Community Survey 5-Year Estimates and Factor Score Coefficients
