# Supplementary material for: Association Between Area-Level Socioeconomic Deprivation and Diabetes Care Quality in US Primary Care Practices
Source: JAMA Netw Open. 2021 Dec 29;4(12):e2138438. doi: 10.1001/jamanetworkopen.2021.38438 (PMC8717098; doi:10.1001/jamanetworkopen.2021.38438)
Supplement: Supplement. — eTable. American Community Survey 5-Year Estimates and Factor Score Coefficients [file jamanetwopen-e2138438-s001.pdf]

Supplementary Online Content

Kurani SS, Lampman MA, Funni SA, et al. Association between area-level socioeconomic deprivation and diabetes care quality in US primary care practices. *JAMA Netw Open*. 2021;4(12):e2138438. doi:10.1001/jamanetworkopen.2021.38438

eTable. American Community Survey 5-Year Estimates and Factor Score Coefficients

This supplementary material has been provided by the authors to give readers additional information about their work.

© 2021 Kurani SS et al. *JAMA Network Open.*

eTable. American Community Survey 5-Year Estimates and Factor Score Coefficients

US Census Indicator	2012-2016 ACS Table Reference, 5-year estimates	Factor Score Coefficient 2016
Median family income	B19013	-0.21740
Income disparity	B19001	0.05598
Families below poverty level	B17010	0.11151
% population below 150% poverty threshold	C17002	0.22992
Single parent household with dependents <18	B23008	0.04159
Households without a motor vehicle	B25044	0.04491
Households without a telephone	B25043	0.01567
Occupied housing units without complete plumbing	B25016	0.01140
Owner occupied housing units	B25003	-0.05758
Households with >1 person per room	B25014	0.01835
Median monthly mortgage	B25088	-0.10626
Median gross rent	B25064	-0.04952
Median home value	B25077	-0.08486
Employed persons ≥16 in white collar occupation	C24010	-0.05700
Civilian labor force unemployed (aged ≥16)	B23025	0.02541
Population aged ≥25 with <9yr education	B15003	0.06044
Population aged ≥25 with at least a high school education	B15003	-0.19303

© 2021 Kurani SS et al. *JAMA Network Open.*
